# Upgrade of the Coherent X-ray Scattering beamline at Pohang Light Source II

**DOI:** 10.1107/S1600577525010367

**Published:** 2026-01-01

**Authors:** Daseul Ham, Daewoong Nam, Changyong Song, Su Yong Lee

**Affiliations:** a Pohang Accelerator Laboratory, POSTECH, Pohang37673, Republic of Korea; b Photon Science Center, POSTECH, Pohang37673, Republic of Korea; c Department of Physics, POSTECH, Pohang37673, Republic of Korea; d Center for Ultrafast Science on Quantum Matter, Max Planck POSTECH/Korea Research Initiative, Pohang37673, Republic of Korea; ESRF – The European Synchrotron, France

**Keywords:** CDI, coherent diffraction imaging, microbeam diffraction, Coherent X-ray Scattering beamline, PLS-II

## Abstract

The Coherent X-ray Scattering beamline at the Pohang Light Source-II was constructed in 2011 for coherent diffraction imaging and has now been upgraded in its focusing optics, diffractometer, detectors and endstation. The enhanced photon flux density and modified endstation have enabled routine Bragg coherent diffraction imaging and microbeam diffraction, while the newly implemented ptychography setup has enhanced nano-imaging capability in transmission geometry. Because coherent diffraction imaging and microbeam diffraction share the same upstream optics, switching between techniques requires only minor adjustments to slit settings, mirror pitch and the sample-to-detector distance, enabling efficient integration of user programs without compromising instrument performance. This paper details the upgrade and the new capabilities of the beamline.

## Abstract 2

Comprehensive upgrades to optics, detectors and the endstation at the CXS beamline have transformed its coherent diffraction imaging performance, enabling routine Bragg coherent diffraction imaging and microbeam diffraction, and adding the capability for transmission geometry nano-imaging via ptychography.

## 1. Introduction

The Coherent X-ray Scattering (CXS) beamline is a hard X-ray undulator beamline located at the 9C port of the Pohang Light Source-II (PLS-II), providing photons in the 5.5–15 keV energy range, and is dedicated to coherent diffraction imaging (CDI) experiments. The beamline was constructed as part of the PLS-II upgrade project (Shin *et al.*, 2013 ▸; Hwang *et al.*, 2014 ▸), which included major improvements such as top-up operation, reduced beam emittance (5.8 nm rad), and increased beam energy (3 GeV) and current (400 mA), all aimed at enhancing storage-ring performance. In establishing the beamline, most of the optics and instruments, including the toroidal mirror, diffractometer and CCD detector, were reused from the 10C1 bending-magnet beamline of the original PLS facility. The vertical beam profile focused by the toroidal mirror deviated from a Gaussian shape and split into multiple distinct lobes owing to gravitational sagging and accumulated surface damage, which severely degraded the photon flux density at the sample. In addition, the CCD detector used for more than ten years became unsuitable for CDI experiments because numerous pixels were permanently damaged and the background level was too high to record weak speckle patterns in the high-*Q* regime. As a result, CDI experiments did not gain momentum even though the photon flux at the sample increased substantially after the introduction of an undulator source in the PLS-II upgrade project.

To make the CXS beamline more favorable to CDI and to establish microbeam diffraction capability, we upgraded the focusing optics, diffractometer, detectors and endstation instruments. The toroidal mirror was replaced with a flat mirror and a Kirkpatrick–Baez (KB) mirror pair. The Eulerian cradle of the standard six-circle diffractometer was replaced with a hexapod motion system. We also introduced photon-counting detectors with a zero-background and a high dynamic range. As a result, CDI, especially Bragg CDI (BCDI), has been invigorated, and the combination of the KB-focused microbeam and improved endstation instruments now enables microbeam diffraction experiments that were previously unavailable to general users at this beamline. Moreover, the recently constructed ptychography setup has enhanced nano-imaging capability in transmission geometry. The following sections describe the upgrade and the resulting improvements in specifications and experimental environments at the CXS beamline.

## 2. Comparison of the beamline layout

**Figure 1:**
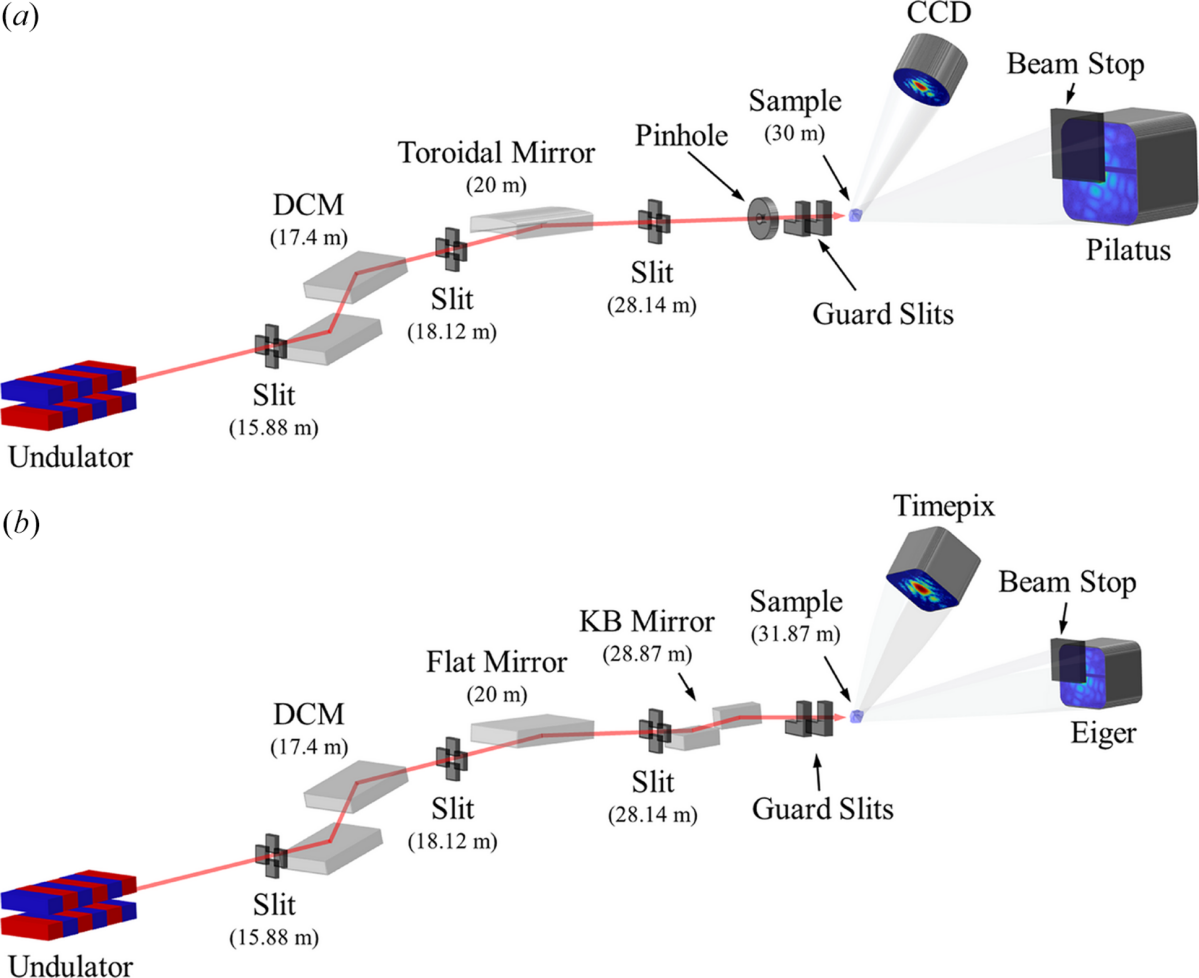
Layout of the CXS beamline (*a*) before and (*b*) after the upgrade.

Fig. 1 ▸(*a*) shows the CXS beamline layout before the upgrade. Distances from the undulator to the major components are indicated in parentheses beneath the component labels. Hard X-rays generated by the undulator were monochromated by an Si(111) double-crystal monochromator (DCM), and the monochromatic beam was focused by a toroidal mirror. The principal parameters of the beamline are described elsewhere (Yu *et al.*, 2014 ▸). A 10 µm-diameter tungsten pinhole and a pair of silicon guard slits were used only for plane-wave CDI (PCDI) experiments (Kim *et al.*, 2020 ▸). These elements were removed from the beam path for BCDI and general diffraction experiments. The Pilatus 1M detector (Dectris, Switzerland) was used for PCDI with a tungsten beam stop, whereas the PIXIS-XO 2048B CCD detector (Teledyne Princeton Instruments, USA) was used for BCDI. General diffraction experiments were performed using a scintillation detector.

After the upgrade, the layout was modified as shown in Fig. 1 ▸(*b*). The toroidal mirror was replaced with a flat mirror and a KB mirror pair. We placed the KB mirror (JTEC Corporation, Japan) with a long focal length of 3 m at the most upstream position of the experimental hutch, slightly increasing the source-to-sample distance from 30 m to 31.87 m. In CDI experiments, the KB-focused beam size is approximately 10 µm, and the transverse coherence length at the sample is approximately 1–3 µm in the 5–9 keV photon energy range used for CDI. Therefore, a tungsten pinhole is not employed, and the silicon guard slits are retained for PCDI only. Two photon-counting detectors were introduced: an Eiger2 X 1M (Dectris, Switzerland) (Donath *et al.*, 2023 ▸) and a Timepix LynX 120 (Amsterdam Scientific Instruments, Netherlands) (Llopart *et al.*, 2007 ▸). The Eiger is used for PCDI, ptychography and microbeam diffraction, whereas the Timepix is used for BCDI.

## 3. Flat mirror and KB mirror

**Figure 2:**
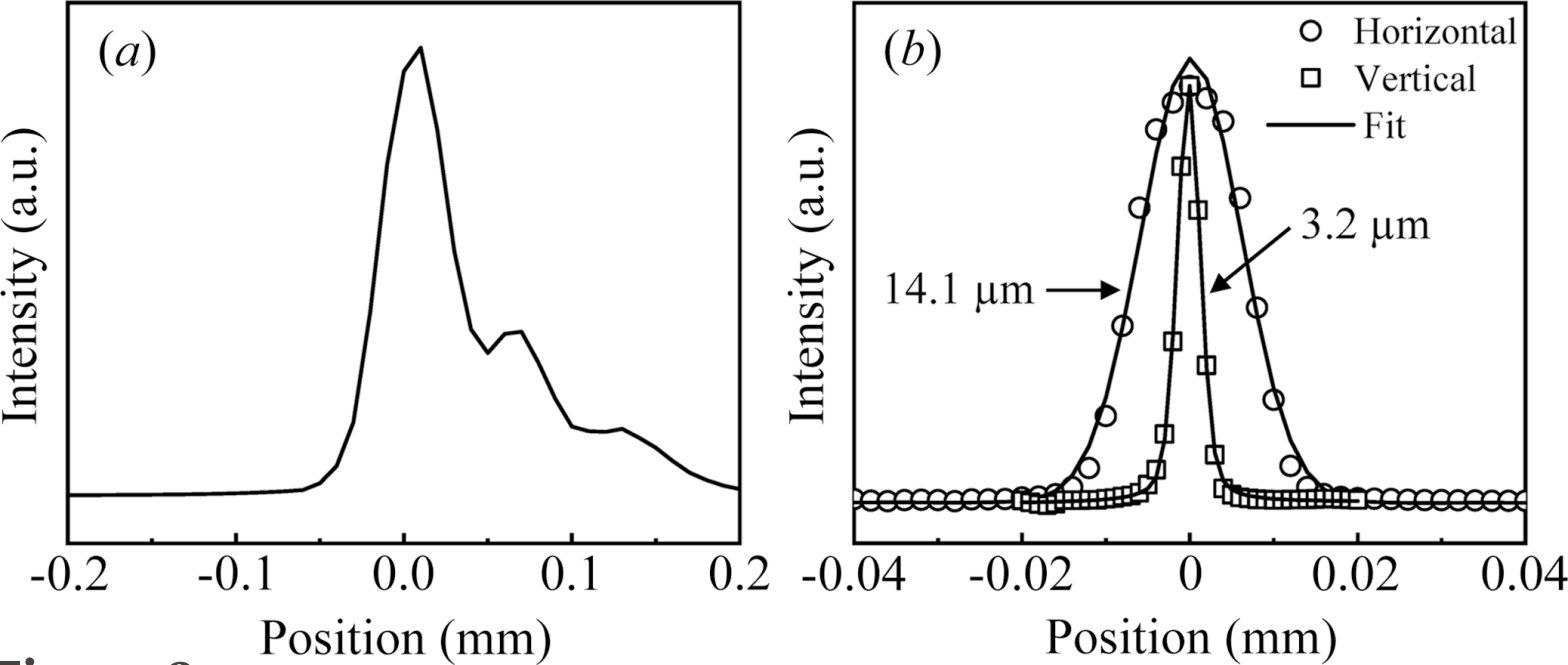
Focused beam profile at the sample position (*a*) before (vertical only) and (*b*) after the upgrade.

Before the upgrade, the most critical issue was the non-ideal focused beam profile at the sample position. The vertical beam profile, measured by scanning a 10 µm-diameter tungsten pinhole, was split into multiple lobes, as shown in Fig. 2 ▸(*a*). This non-ideal profile was attributed to the toroidal mirror, whose surface profile had been degraded by gravitational sagging and repeated bending/unbending motions. In CDI, the typical particle size is about 500 nm, which is smaller than the focused beam. In this case, the measured diffraction intensity is influenced by the photon flux density rather than by the total flux, because only photons incident on the sample contribute. A multi-lobed focus distributes the available flux into separate intensity peaks and therefore lowers the local flux density at the sample when the particle is centered on one peak, which diminishes the diffracted intensity. Improving the focused beam profile increases the flux density at the sample, thereby enabling the acquisition of speckle patterns up to the high-*Q* range for high-resolution imaging. We therefore replaced the toroidal mirror with a flat mirror and a KB mirror pair. Specifications of the flat mirror and the KB mirror are summarized in Table 1 ▸. For the KB mirror, the tangential slope error and the surface roughness were specified to be below 0.1 µrad r.m.s. and 0.2 nm r.m.s., respectively, to achieve an intense Gaussian beam. The incidence angle was set to 3 mrad. Because the slit immediately upstream of the KB mirror is typically set to no more than 250 µm to satisfy the transverse coherence requirement, the total flux of the focused beam is limited by the upstream slit rather than by the clear aperture of the mirror. We note that, for photon energies below 6 keV, we use the bare Si surface of the flat mirror together with second crystal detuning of the DCM to suppress higher harmonics in PCDI and ptychography.

In the new mirror system, a virtual source is defined in the horizontal direction by a slit (15.88 m from the undulator) installed upstream of the DCM, because the undulator source size is relatively large horizontally as indicated in Table 5. The slit opening is typically set to 70 µm. The focused beam profile at the sample, measured by knife-edge scans, is shown in Fig. 2 ▸(*b*). The focal spot size (FWHM) is 3.2 µm in the vertical direction and 14.1 µm in the horizontal direction. The vertical size is the minimum attainable and is limited by source demagnification, whereas the horizontal size can be further reduced by decreasing the horizontal source size, at the expense of beam intensity. For CDI experiments, we increase the vertical beam size by adjusting the pitch of the vertical KB mirror so that the transverse coherence length at the sample exceeds at least twice the sample size (Spence *et al.*, 2004 ▸). At 8 keV, the transverse coherence length, defined as λ*R*/2*D* (Als-Nielsen & McMorrow, 2011 ▸), where λ is the X-ray wavelength, *R* is the distance from the source and *D* is the source size, was calculated to be 34.5 µm in the vertical direction and 14.4 µm in the horizontal direction at the slit immediately upstream of the KB mirror. The ratio of the transverse coherence length to the slit opening is preserved at the focus (Vartanyants & Singer, 2010 ▸). With the slit immediately upstream of the KB mirror set to 200 µm, the transverse coherence length at the sample is 0.6 µm in the vertical direction and 1.0 µm in the horizontal direction for the focused beam shown in Fig. 2 ▸(*b*). Such a vertical coherence length is insufficient for reliable phase retrieval of typical CDI samples (approximately 500 nm). Increasing the vertical beam size to 8 µm raises the vertical coherence length to 1.4 µm. The transverse coherence length in both directions can be extended beyond 3 µm by lowering the photon energy to 5.55 keV, reducing the horizontal source size to 50 µm, and narrowing the slit at the KB mirror to 100 µm.

## 4. Modified six-circle diffractometer

**Figure 3:**
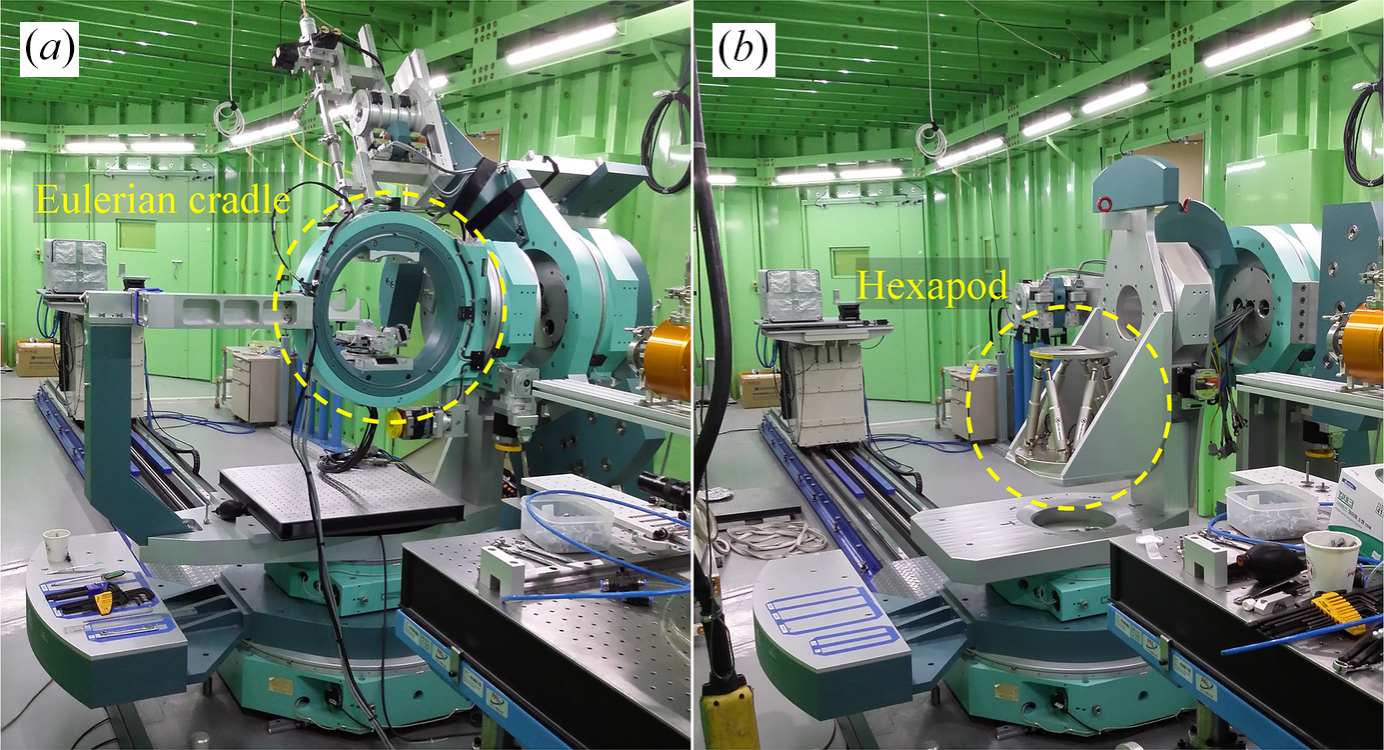
Photographs of (*a*) the standard six-circle diffractometer and (*b*) the modified six-circle diffractometer with the hexapod motion system and L-shaped bracket.

The standard six-circle diffractometer with the Eulerian cradle shown in Fig. 3 ▸(*a*) is well suited to measuring in-plane diffraction, and strain tensors can be deduced from several in-plane reflections in BCDI (Hofmann *et al.*, 2020 ▸; Wilkin *et al.*, 2021 ▸). However, installation of an *in situ* chamber is severely limited by the small diameter of the chi-circle (approximately 360 mm) in the Eulerian cradle. Because most users primarily measure out-of-plane diffraction and prefer *in situ* or *operando* studies over full strain-tensor analyses, we replaced the Eulerian cradle with the H-840 hexapod motion system (Physik Instrumente, Germany) mounted on the L-shaped bracket, as shown in Fig. 3 ▸(*b*). Specifications of the sample-related motions provided by the hexapod are summarized in Table 2 ▸. Although in-plane diffraction is more limited in the modified diffractometer due to the reduced travel range of the chi- and phi-circles, the hexapod provides clear advantages: (i) mounting heavy or bulky apparatus is facilitated because the load capacity increased to 30 kg (from 10 kg) and the small chi-circle was removed; (ii) chamber design is facilitated by a 100 mm clearance between the sample and the top surface of the motion system (this clearance, equal to the distance from the rotation center of the diffractometer to the top surface of the hexapod, was 70 mm previously); and (iii) rotating the theta-circle by 180° provides an available space of 500 mm (W) × 825 mm (D) × 530 mm (H) on the diffractometer, allowing large custom experimental setups to be mounted. Removing the L-shaped bracket together with the hexapod increases the available space to 500 mm × 825 mm × 1040 mm.

## 5. Photon-counting detectors

**Figure 4:**
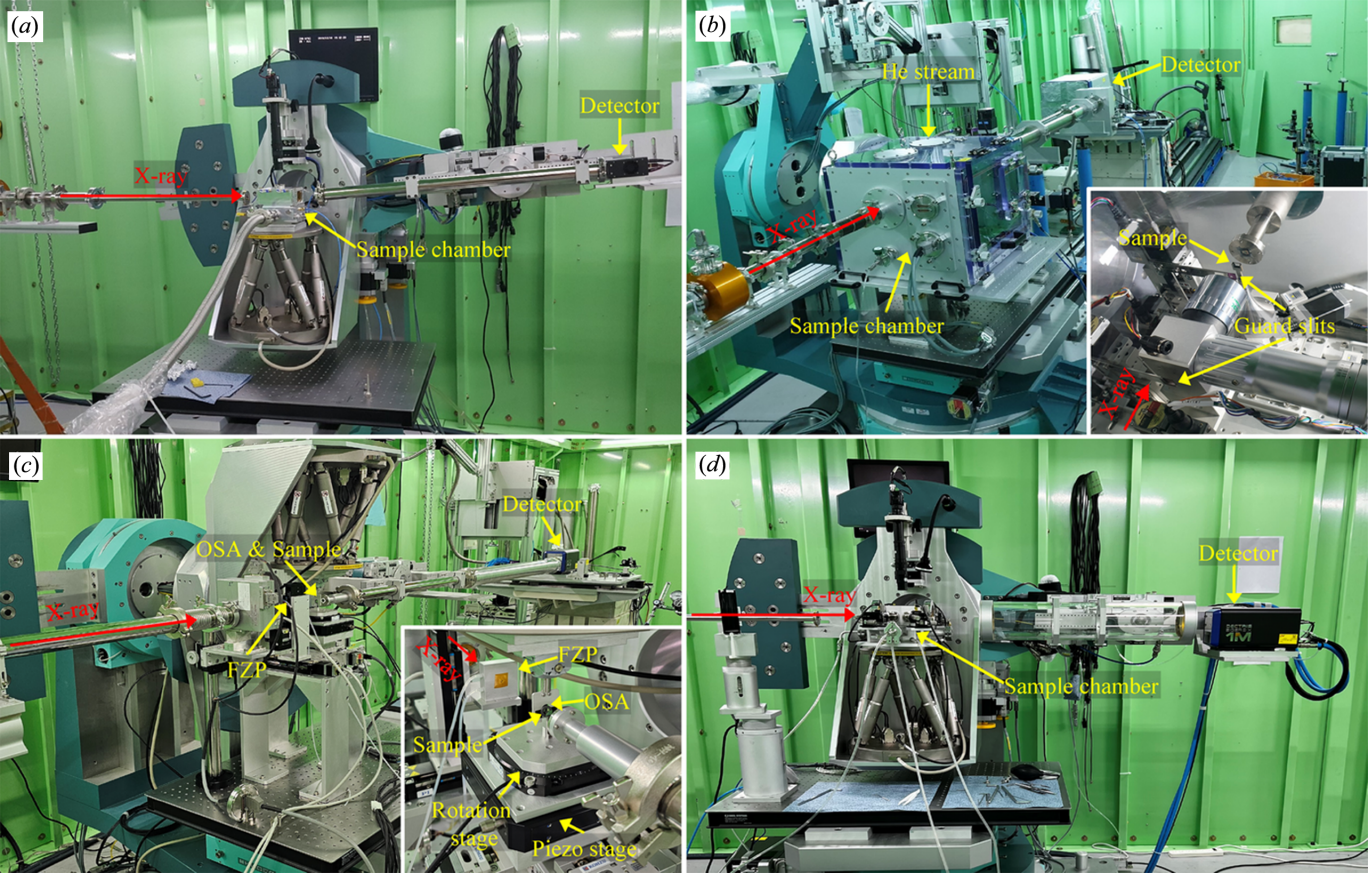
Endstation setups for (*a*) BCDI, (*b*) PCDI, (*c*) ptychography and (*d*) microbeam diffraction.

The PIXIS-XO 2048B CCD detector, utilized in CDI experiments for over a decade prior to the beamline upgrade, had accumulated numerous defective pixels resulting from exposure to the direct X-ray beam. Furthermore, its high inherent background noise (dark current) was unsuitable for these experiments, as it is detrimental to the recording of weak speckle patterns in the high-*Q* regime. We therefore introduced two photon-counting pixel-array detectors with zero-background noise: a Timepix LynX 120 and an Eiger2 X 1M. Specifications of the two detectors are summarized in Table 3 ▸. The small 55 µm pixel size of the Timepix detector allows an oversampling ratio greater than 2, and its compact form factor eases adjustment of the sample-to-detector distance (SDD). Accordingly, it is used for BCDI. Despite the modest maximum counts per pixel (11810), it is suitable for BCDI because the diffraction intensity from a single particle of crystalline material 300 to 1500 nm in size falls within this range. The Eiger detector is used for PCDI, ptychography and microbeam diffraction, where strong diffraction patterns are recorded. For microbeam diffraction it is mounted on the detector arm of the diffractometer, and for PCDI and ptychography it is placed on a motorized detector table, as shown in Fig. 4 ▸.

## 6. Endstation setup for CDI and microbeam diffraction

The modified six-circle diffractometer facilitates mounting heavy apparatus and provides sufficient space for customized chamber systems. Fig. 4 ▸(*a*) shows the BCDI setup. An *in situ* chamber capable of heating to 550°C is mounted on the hexapod. The chamber also allows atmosphere control, including evacuation and inert-gas purging. For *operando* imaging of lithium-ion battery samples, a coin-cell holder and an AMPIX cell (Borkiewicz *et al.*, 2012 ▸) are available. The coherent diffraction pattern is recorded with the Timepix detector mounted on the detector arm of the diffractometer, and the SDD is adjusted manually between 0.7 m and 1.5 m depending on photon energy and sample size. The length of the evacuated beam path between the sample and the detector is adjusted by assembling NW40 spools. Both ends are sealed with 50 µm-thick Kapton films.

Fig. 4 ▸(*b*) shows the PCDI setup. To install the massive chamber, which is capable of imaging biological samples, the L-shaped bracket and the hexapod motion system are removed from the diffractometer. A pair of guard slits for eliminating parasitic scattering and a sample manipulator with step-motor-driven *XYZ* and rotation stages are mounted inside the chamber. A retractable microscope, whose field of view is centered on the X-ray beam at the sample, is positioned between the downstream guard slit and the sample. The chamber is purged with helium to minimize absorption and elastic scattering. A 100 K helium stream is applied only when imaging biological samples, directed downwards immediately above the sample. The coherent diffraction pattern propagates through an evacuated beam path sealed with a 1 µm-thick silicon nitride membrane at the upstream end and a 50 µm-thick Kapton film at the downstream end, and is recorded by the Eiger detector. A 4 mm-thick tungsten beam stop is used to protect the detector from the direct X-ray beam, producing the shadowed region around the direct beam as shown in Figs. 6(*a*) and 6(*b*). The upstream silicon nitride membrane is employed to minimize small-angle scattering background, whereas the downstream Kapton film is used to ensure mechanical robustness and a large clear aperture near the detector. A high-dynamic-range detector is essential in PCDI because intense parasitic scattering is recorded together with the speckle pattern despite the guard slits.

Fig. 4 ▸(*c*) shows the ptychography setup in transmission geometry. Because the setup is compact, the theta-circle is rotated by 180° rather than removing the L-shaped bracket and the hexapod motion system. The KB mirror is bypassed since focusing is provided by a tungsten Fresnel zone plate (FZP) with a 300 µm diameter, 60 nm outermost zone width, 1 µm thickness and a focal length of 123 mm at 8.5 keV. The typical probe size at the sample is approximately 500 nm. A platinum central stop (CS; 50 µm diameter, 50 µm thickness) is fixed on the FZP assembly. The FZP is housed in a compact helium-purged chamber and translated by a step-motor-driven *XY* stage. A tungsten order-sorting aperture (OSA; 30 µm diameter, 25.4 µm thickness) is mounted on the hexapod and positioned 5 mm upstream of the focal plane of the FZP. The sample manipulator comprises a step-motor-driven *XYZ* stage, a P-561 piezoelectric *XYZ* stage (Physik Instrumente, Germany) and a U-651 piezoelectric rotation stage (Physik Instrumente, Germany), and is positioned at the focal plane of the FZP. Considering the transverse coherence length, the size of the X-ray beam is defined to be about 40 µm by a slit upstream of the FZP. The incident X-ray beam (∼40 µm) is not aligned to the center of the FZP, as the 50 µm CS completely obstructs the beam. Instead, the beam is positioned towards the periphery of the FZP, which enables effective focusing while mitigating the issue of an excessively large effective outermost zone width arising from the limited beam size. A 65 mm^2^ silicon drift detector (Rayspec, UK) with an Xspress 3 Mini pulse processor (Quantum Detectors, UK) can also be installed upon request for chemical analysis. The sample is raster-scanned at the FZP focus while recording the coherent diffraction pattern and X-ray fluorescence at each position. The coherent diffraction pattern propagates through the evacuated beam path described for the PCDI configuration (1 µm-thick silicon nitride entrance window and 50 µm-thick Kapton exit window) and is recorded with the Eiger detector. Unlike the PCDI setup, the evacuated beam path is mounted on the detector arm of the diffractometer rather than the floor, simplifying installation.

**Figure 5:**
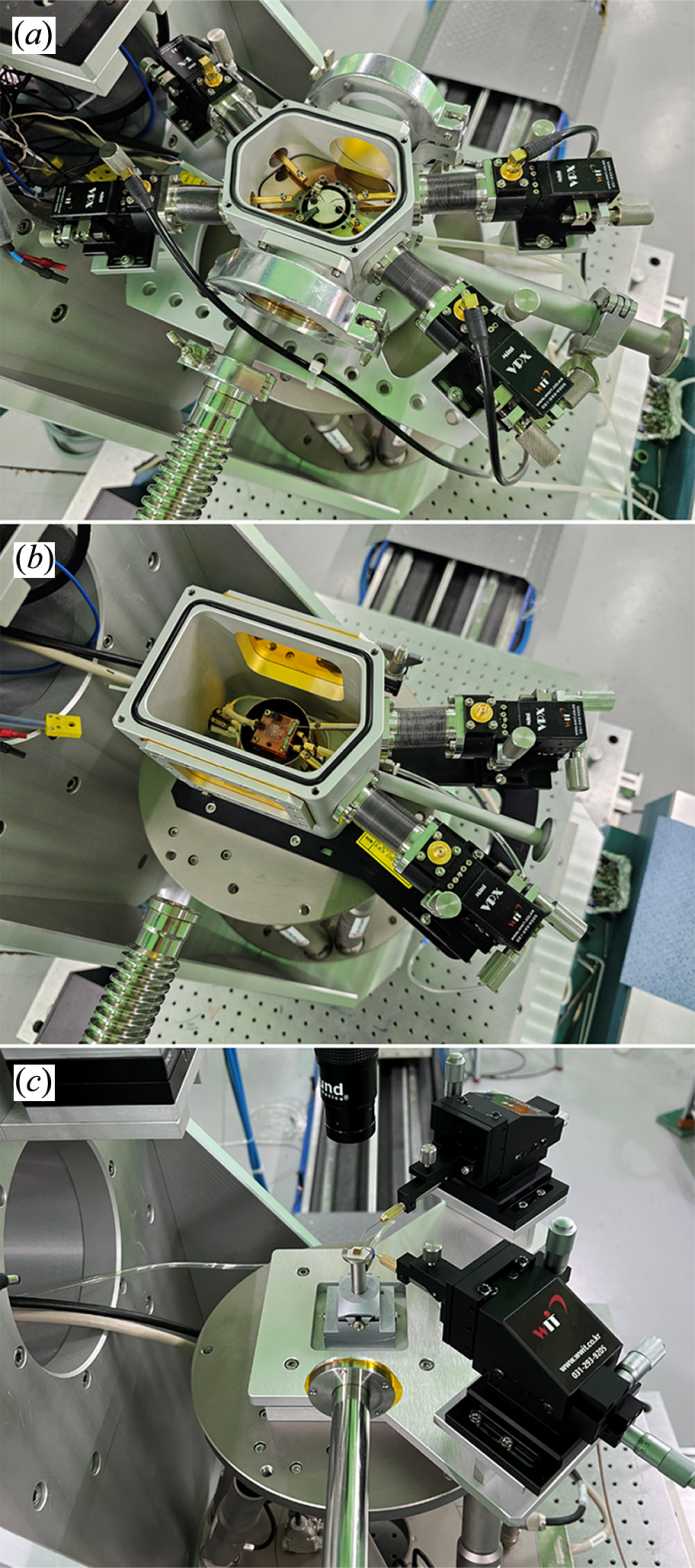
Photographs of the probe systems: (*a*) four-probe station chamber, (*b*) two-probe station chamber and (*c*) two-probe positioner system.

Fig. 4 ▸(*d*) shows the microbeam diffraction setup, which is identical to the BCDI setup except that an evacuated beam path of 100 mm diameter is mounted on the detector arm and intense diffraction is recorded with the Eiger detector. Microbeam diffraction at the CXS beamline is specialized for time-resolved diffraction and multimodal analysis with probe systems such as a four-probe station chamber (Ham *et al.*, 2022 ▸), a two-probe station chamber and a two-probe positioner system. Although the probe systems were devised for time-resolved diffraction and multimodal analysis, they can also be used in BCDI. In time-resolved diffraction, an electric field pump is applied to the sample (*e.g.* ferroelectric thin films) via a pair of tungsten probe tips, and structural changes are probed by X-ray diffraction. Details of the experimental sequence are described elsewhere (Choi *et al.*, 2024 ▸). Because only two probe tips are required for field application, any of the three probe systems can be used. In multimodal analysis, the probe tips are used to measure electrical properties such as resistance while X-ray diffraction is recorded simultaneously (Choi *et al.*, 2025 ▸). The two-probe station chamber or the two-probe positioner system is used for two-wire measurements, whereas the four-probe station chamber is used for four-wire measurements. Fig. 5 ▸ shows photographs of the probe systems. Their specifications are summarized in Table 4 ▸.

## 7. Highlights

**Figure 6:**
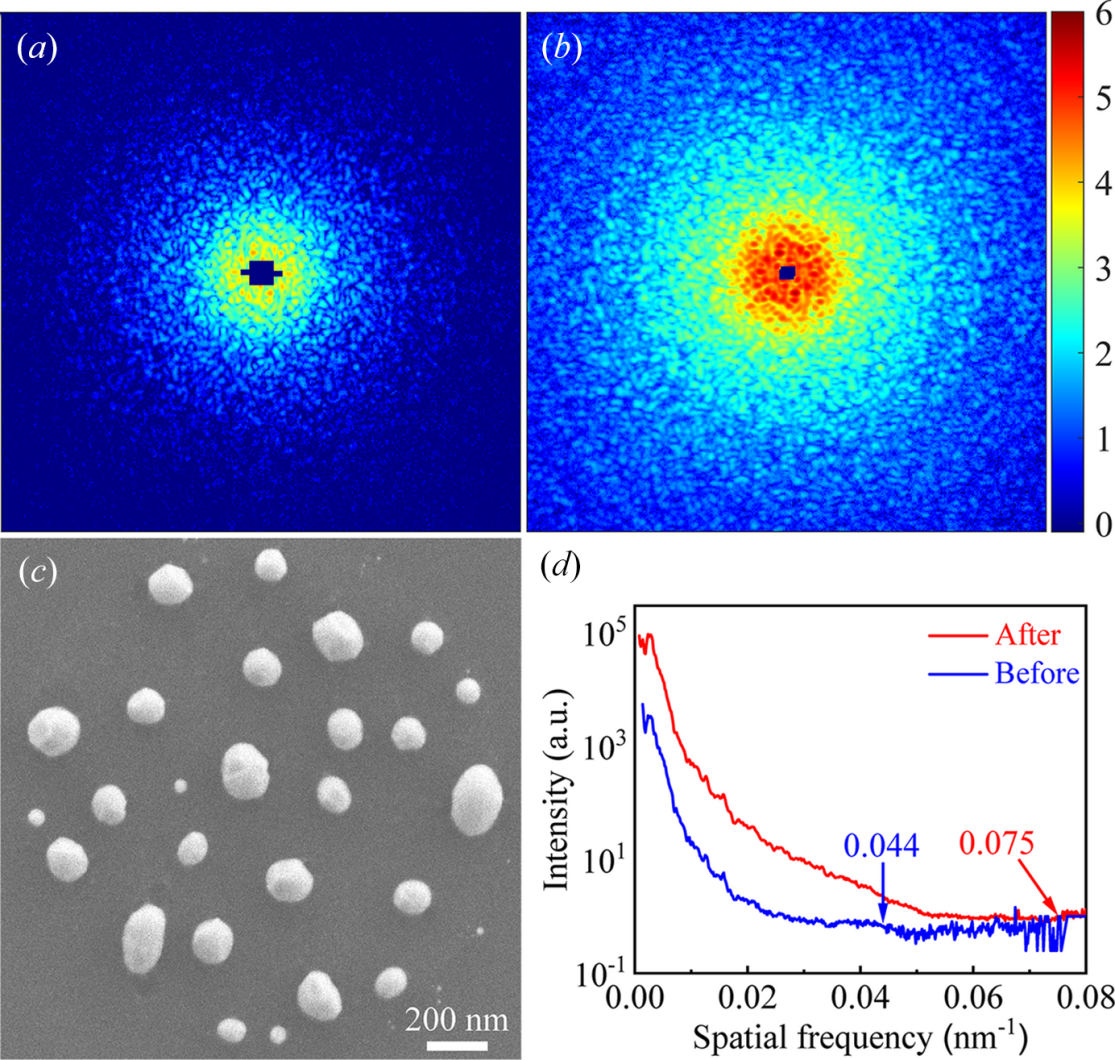
Log-scale speckle patterns from the gold-nanoparticle test sample (*a*) before and (*b*) after the upgrade. (*c*) Scanning electron microscopy image of the test sample. (*d*) PSD of the two speckle patterns.

Table 5 ▸ lists the input conditions for the *OASYS* simulation (Rio & Rebuffi, 2019 ▸) and the calculated photon flux densities, indicating a ∼17-fold increase after the upgrade. Performance enhancement was evaluated using the PCDI setup with a gold-nanoparticle test sample [Fig. 6 ▸(*c*)] fabricated as described elsewhere (Kim *et al.*, 2017 ▸). PCDI is inherently robust to sample vibration and drift (Miao *et al.*, 2015 ▸) and is therefore suitable for examining the enhancement of the photon flux density at the sample. Figs. 6 ▸(*a*) and 6 ▸(*b*) show log-scale speckle patterns from the test sample before and after the beamline upgrade. To acquire these patterns, the major parameters were kept constant, with the photon energy at 5.6 keV and the exposure time at 3 min. The SDDs differed slightly, 4990 mm before the upgrade and 4930 mm after the upgrade. To quantify the improvement, we computed the power spectral density (PSD) of the two speckle patterns as depicted in Fig. 6 ▸(*d*). The cutoff spatial frequency, beyond which diffraction signals become indistinguishable from noise, increased from 0.044 nm^−1^ (22.7 nm) to 0.075 nm^−1^ (13.3 nm) with the beamline upgrade.

**Figure 7:**
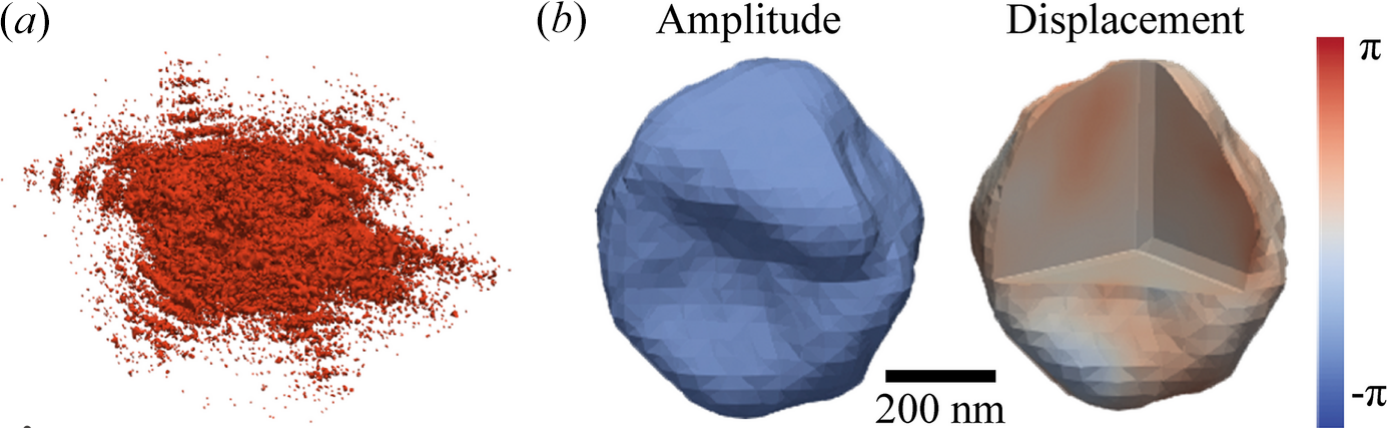
(*a*) 3D diffraction pattern around the LiMn_2_O_4_ (004) Bragg peak and (*b*) reconstructed 3D images.

With the enhanced coherent flux and the adoption of high-performance photon-counting detectors, BCDI studies of lithium-ion battery materials have increased at the CXS beamline. Fig. 7 ▸ shows a 3D diffraction pattern around the LiMn_2_O_4_ (004) Bragg peak and the corresponding 3D reconstructions obtained using error-reduction and hybrid input–output algorithms (Clark *et al.*, 2015 ▸; Clark *et al.*, 2012 ▸). For *in situ* or *operando* experiments, in addition to the coin-cell holder and the AMPIX cell, we have prepared an ultrasonic cleaner and a spin coater for particle dispersion, a heating chamber, and battery cyclers such as the WBCS3000Le (Wonatech, Korea) and the SP-200 (Biologic, France). We note that the accessible sample size in BCDI is on the order of 300–1500 nm. The upper limit is primarily constrained by the transverse coherence length but can be reduced, depending on photon energy, to ensure an oversampling ratio greater than 3.1 for best reconstruction quality (Öztürk *et al.*, 2017 ▸). The lower limit is constrained by the photon flux density at the sample.

**Figure 8:**
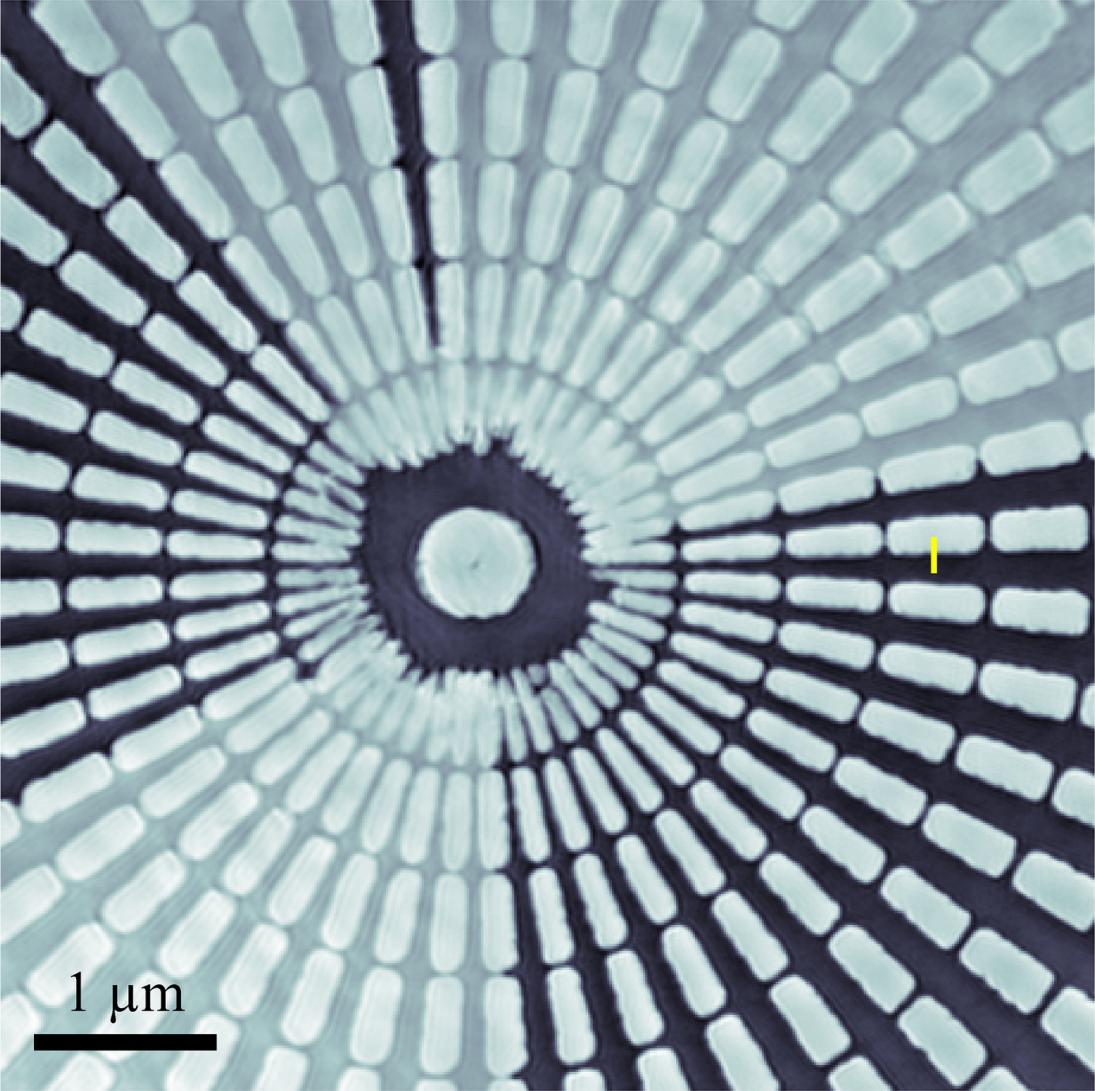
Phase image of a Siemens-star pattern obtained using the ptychography setup (innermost period: 80 nm).

**Figure 9:**
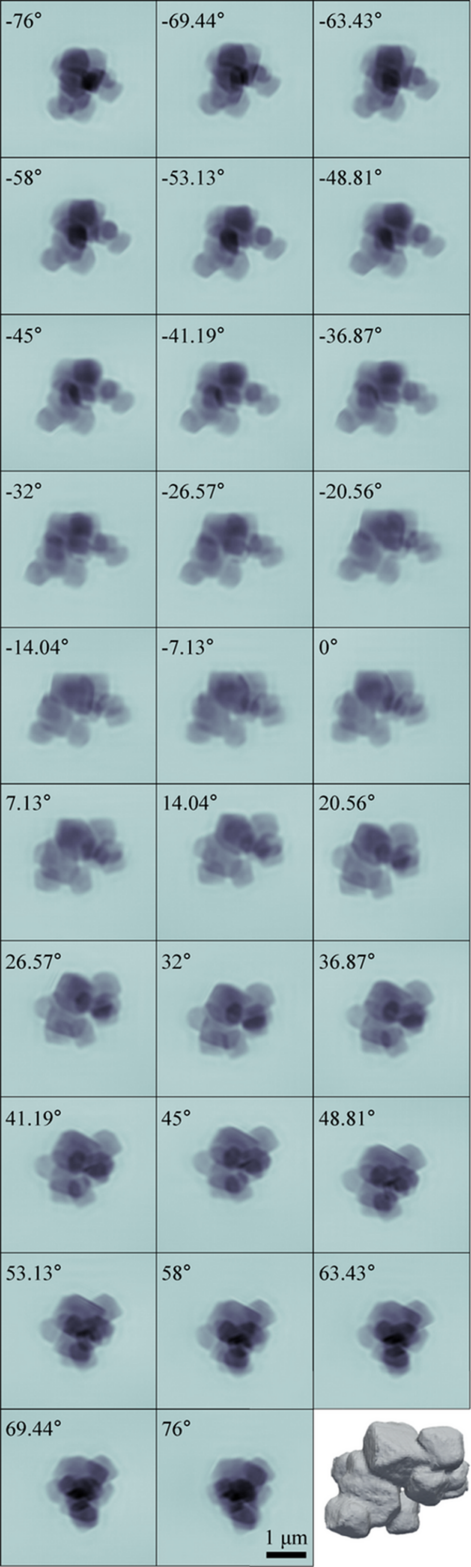
Ptychographic tomography of LiNiCoMnO_2_ (NCM 111) cathode particles.

A ptychography setup was recently constructed to enable high-resolution imaging of extended samples, thereby further enhancing the imaging capability of the CXS beamline. Fig. 8 ▸ shows a phase image of a Siemens-star pattern obtained at 8.5 keV with an SDD of 2000 mm. The X-ray probe size was approximately 500 nm, and the sample was raster-scanned with a step size of 200 nm and an exposure time of 0.5 s. For routine imaging with less demanding resolution or stronger scatterers, exposure times shorter than 0.5 s are feasible. Ptychographic reconstruction was performed using the *PtychoShelves* package (Wakonig *et al.*, 2020 ▸). The reconstructed pixel size was 13.0 nm, and the knife-edge-based spatial resolution was estimated to be 34.7 nm. The innermost features of the pattern, corresponding to an 80 nm period, were clearly resolved. The alternating black and gray regions also visible in the phase image are attributed to accumulated sample damage and fabrication imperfections. Fig. 9 ▸ shows a ptychographic tomography dataset of LiNiCoMnO_2_ (NCM 111) cathode particles. Ptychographic phase images were acquired at sample rotation angles from −76° to +76°, and the 3D phase volume was reconstructed from these phase maps using the *RESIRE* code (Pham *et al.*, 2023 ▸). The voxel size was (32.5 nm)^3^, and the effective resolution, estimated from the Fourier shell correlation (Rosenthal & Henderson, 2003 ▸) using the 0.143 criterion, was 70.4 nm. For transmission geometry imaging at the CXS beamline, PCDI is efficient for rapid 2D imaging of isolated samples because it does not require lateral scanning. Data acquisition is typically completed within a few minutes, which is advantageous for *in situ*/*operando* monitoring. By contrast, ptychography requires a lateral scanning and typically takes 10–20 min for a modest 2D imaging, but it accommodates extended samples and can be combined with X-ray fluorescence microscopy to provide chemical analysis. For static or slowly evolving extended samples, ptychography is particularly effective and can be used for high-resolution 3D imaging.

## 8. Conclusions

We have upgraded the focusing optics, diffractometer, detectors and endstation instruments at the CXS beamline. The enhanced photon flux density, together with the modified endstation, have invigorated BCDI and enabled microbeam diffraction. The implementation of a ptychography setup has strengthened nano-imaging capability in transmission geometry. An important outcome of the upgrade is that CDI and microbeam diffraction can now be operated on the same beamline using shared upstream beam optics, namely the undulator, the DCM, a flat mirror and KB mirrors. Switching between techniques requires only minor adjustments to the slit settings, the mirror pitch and the SDD, enabling efficient integration of user science programs without compromising instrument performance. To streamline operations further, we plan to install a motorized stage on the detector arm of the diffractometer for automated SDD control and to redesign the ptychography setup so that the FZP and the sample scanner are mounted on a unified base to simplify installation.

## Figures and Tables

**Table 1:** Specifications of the flat mirror and the KB mirror

Parameter	Flat	KBV	KBH
Shape	Flat	Elliptical	Elliptical
Substrate material	Si	Si	Si
Coating	Rh	Rh	Rh
Mirror length (mm)	800	150	150
Tangential slope error (µrad rms)	1.25	0.1	0.1
Sagittal slope error (µrad rms)	10	1	1
Roughness (nm rms)	0.3	0.2	0.2
Incidence angle (mrad)	4.2	3	3

**Table 2:** Specifications of the sample-related motions provided by the hexapod *X* is parallel to the floor, *Y* is toward the ceiling and *Z* is along the beam propagation direction. Travel range for each axis indicates its maximum travel range when all other axes are in the zero position.

Parameter	Value
Travel range in *X*, *Z* (mm)	±50
Travel range in *Y* (mm)	±25
Travel range in Chi (°)	±15
Travel range in Phi (°)	±30
Minimum incremental motion in *X*, *Z* (µm)	0.3
Minimum incremental motion in *Y* (µm)	0.2
Minimum incremental motion in Chi (µrad)	3
Minimum incremental motion in Phi (µrad)	5
Repeatability in *X*, *Z* (µm)	±0.3
Repeatability in *Y* (µm)	±0.15
Repeatability in Chi (µrad)	±2.5
Repeatability in Phi (µrad)	±3

**Table 3:** Specifications of the photon-counting detectors For the Timepix detector, the maximum count rate equals the counter depth (11810 photons frame^−1^ pixel^−1^) multiplied by the maximum frame rate, and data should be acquired so that the per-pixel counts remain below this limit.

Parameter	TimepixLynX120	Eiger2X1M
Sensor	Si	Si
Sensor thickness (µm)	300	450
Pixel size (µm)	55	75
Number of pixels (W×H)	512×512	1028×1062
Active area (mm × mm) (W×H)	28.2×28.2	77.1×79.7
Maximum count rate (photons s^−1^ pixel^−1^)	1417200	9562500
Maximum frame rate (Hz)	120	4500 (8-bit)
		2250 (16-bit)

**Table 4:** Specifications of the probe systems

Parameter	Four-probe station chamber	Two-probe station chamber	Two-probe positioner system
Temperature range (°C)	RT–550	RT–550	RT only
Maximum angle (2θ, °)	45	80	Not limited
Sample size (mm)	15 × 15	15 × 15	Not limited

**Table 5:** Positions of the major components from the undulator used for the *OASYS* simulation and the calculated photon flux density at 5.6 keV Values in parentheses denote the X-ray beam size (FWHM) or slit opening.

	Source	Slit	DCM	M1	Slit	Focusing optics	Sample	Flux density (photons s^−1^ µm^−2^) at sample (5.6 keV)
Before upgrade (V×H, µm)	0 m (27×423)	15.88 m (200×70)	17.4 m	–	19.2 m (200×200)	20 m (toroidal)	30 m (14.1×195.4)	1.05×10^7^
After upgrade (V×H, µm)	0 m (27×423)	15.88 m (200×70)	17.4 m	20 m (flat)	28.5 m (KBV)	28.7 m (KBV)	31.87 m (3.1×15.2)	1.79×10^8^
						28.87 m (KBH)		
